# Neoadjuvant Therapy and Factors Influencing Survival in Locally Advanced Non-Small Cell Lung Cancer

**DOI:** 10.7759/cureus.33392

**Published:** 2023-01-05

**Authors:** Senar Ebinç, Zeynep Oruç, Ziya Kalkan, Fatma Teke, Serdar Onat, Zuhat Urakçı, Muhammet Ali Kaplan, Mehmet Küçüköner, Abdurrahman Işıkdoğan

**Affiliations:** 1 Department of Medical Oncology, Gazi Yasargil Training and Research Hospital, Diyarbakır, TUR; 2 Department of Medical Oncology, Dicle University Faculty of Medicine, Diyarbakır, TUR; 3 Department of Radiation Oncology, Dicle University Faculty of Medicine, Diyarbakır, TUR; 4 Department of Thoracic Surgery, Dicle University Faculty of Medicine, Diyarbakır, TUR

**Keywords:** multimodal treatment, surgery, chemoradiotherapy, lung cancer, neoadjuvant chemotherapy

## Abstract

Aim: We aimed to investigate the effectiveness of neoadjuvant therapy (NAT) and clinicopathological characteristics in locally advanced non-small cell lung cancer (NSCLC) (IIIA-IIIB), as well as the influence of the post-NAT treatment modalities on survival.

Materials and methods: This study included patients who presented to the Dicle University Medical Oncology Clinic and received NAT for a diagnosis of locally advanced NSCLC between 2004 and 2020. Clinicopathological and radiological data of the 57 patients whose data could be retrieved from the hospital archive system were retrospectively reviewed. Patients’ overall survival (OS) and failure-free survival (FFS) times and the factors influencing these times were evaluated.

Results: This study included a total of 57 patients consisting of five (8.8%) females and 52 (91.2%) males. The median patient age at diagnosis was 58 (30-75) years. All patients had received four courses of chemotherapy during the neoadjuvant period. When the factors influencing OS were evaluated, the post-NAT modality was found to have a statistically significant effect on survival. FFS times were 12, 13, and 16 months in the chemotherapy, chemoradiotherapy, and surgery arms, respectively (log-rank p=0.035). FFS was longer in those who underwent surgery (Hazard ratio (HR): 0.33, 95 % CI: 0.14-0.77, (p=0.01)). OS times were 20, 21, and 55 months in the chemotherapy, chemoradiotherapy, and surgery arms, respectively (log-rank p=0.05). OS was longer in the arm undergoing surgery compared to the other arms (HR: 0.36, 95% CI: 0.14-0.87, (p=0.02)). Five-year survival rates for the chemotherapy, chemoradiotherapy, and surgery arms were 14.3%, 21.4%, and 40%, respectively.

Conclusions: This study shows that achieving an operable status is the most important indicator of survival and that patients undergoing surgery have a marked advantage in OS and FFS compared with patients receiving chemoradiotherapy or palliative chemotherapy.

## Introduction

Lung cancer is among the most important causes of cancer-related deaths [1]. The surgical approach alone is insufficient in locally advanced (IIIA-N2) non-small cell lung cancer (NSCLC) [2]. In this stage, systemic treatments are discussed with respect to the control of both systemic micrometastases and local control. Neoadjuvant therapies (NATs) offer an advantage over adjuvant treatment due to the control of systemic spread in the preoperative period, the patient being able to receive the complete treatment as a result of better patient performance, better tumor resectability, and the opportunity to evaluate treatment response more objectively [3-5]. Although the studies that have investigated the benefits of induction chemotherapy in comparison with the surgical approach alone reported conflicting results, NAT was shown to offer a survival benefit in a study by the Collaborative Group [5-7]. Induction chemotherapy was reported to offer better outcomes for superior sulcus tumors compared with chemotherapy, with no additional benefit for tumors in other localizations [8,9]. There are also studies reporting that cisplatin + docetaxel in induction therapy prior to chemotherapy is tolerable in locally advanced NSCLC, while some studies have described that cisplatin + docetaxel in induction therapy yields better outcomes than cisplatin + gemcitabine therapy [10,11]. In this study, we aimed to investigate the effectiveness of NAT and the clinicopathological characteristics in our patients diagnosed with locally advanced (IIIA-IIIB) NSCLC, as well as the effects of the treatment modalities applied in the period following NAT on survival.

## Materials and methods

Our study included patients older than 18 years who presented to the Dicle University Medical Oncology Clinic and received NAT for a diagnosis of locally advanced NSCLC between 2004 and 2020. Data from a total of 57 patients could be retrieved. Clinicopathological and radiological data of the patients from the hospital archive system were retrospectively reviewed. Patients' gender, age, smoking status, initial stage, histological tumor subtype, neoadjuvant chemotherapy regimen, response to NAT, post-NAT treatment modalities, and recurrence or progression states were recorded. Patients’ overall survival (OS) and failure-free survival (FFS) times and the factors influencing these times were evaluated. The effects of post-NAT treatment modalities on survival times were investigated.

Patient inclusion and exclusion criteria

This study included patients diagnosed with NSCLC who were in a locally advanced stage (IIIA (T1-2, N2), IIIB (T3, N2)) with an indication for NAT. Tumor staging was performed in accordance with the American Joint Committee on Cancer (AJCC) version 8 tumor, node, metastasis (TNM) staging system by retrospectively using radiological data from the hospital archive system. Metastatic and oligometastatic patients were not included.

Treatments, definitions, and response evaluation

As NAT, four cycles of cisplatin 75 mg/m^2^ (1st day) + paclitaxel 175 mg/m^2^ (1st day) or cisplatin 75 mg/m^2^ (1st day) + gemcitabine 1250 mg/m^2^ (1st and 8th days) or cisplatin 75 mg/m^2^ (1st day) + docetaxel 75 mg/m^2^ (1st day) were administered in the form of 21-day cycles. In patients who were not suitable for surgery after NAT, weekly paclitaxel 45-50 mg/m^2^ + carboplatin area under the curve (AUC) 2 or paclitaxel 45-50 mg/m^2^ + cisplatin 25 mg/m^2^ were administered concurrently with 60 Gy radiotherapy in 30 fractions. In operated patients, lobectomy or pneumonectomy was performed. In surgery-arm patients with margin positivity or N2 disease after surgery, adjuvant radiotherapy was administered. Meanwhile, patients not suitable for chemoradiotherapy or surgery after NAT were initiated on second-line chemotherapy (chemotherapy arm) in the presence of progression. Response after NAT was evaluated by computerized tomography or positron emission tomography based on Response Evaluation Criteria in Solid Tumours (RECIST) v 1.1. The period of time from the diagnosis to death was calculated as the OS and the period of time from the end of NAT to recurrence or progression as FFS.

Statistical analysis

The Predictive Analytics SoftWare (PASW) Statistics for Windows, Version 18.0. (SPSS Inc., Chicago, USA) software was used for statistical analysis. Patient characteristics and the frequency of parameters were evaluated using descriptive statistics and the distribution of parameters across groups using one-way analysis of variance (ANOVA). In survival analyses, the Cox regression analysis - Enter method was used for univariate analysis, and the Cox regression analysis - Backward stepwise (likelihood ratio) method was used for multivariate analysis. The Kaplan-Meier method was used to perform survival analyses. The log-rank p-value was considered the basis. A confidence interval (CI) of 95% and a two-tailed significance value of p<0.05 were taken.

Ethical approval

It was obtained from the Dicle University Faculty of Medicine Ethics Committee (date/number: 12.05.2022/131).

## Results

Our study included a total of 57 patients consisting of five (8.8%) females and 52 (91.2%) males. The median patient age at diagnosis was 58 (30-75) years. As the histological subtype, 19 (33.3%) patients had adenocarcinoma, and 38 (66.7%) patients had squamous cell carcinoma histology. History of smoking was positive in 96.5% (n=55) of the patients. At diagnosis, 41 (71.9%) patients had stage 3A disease and 16 (28.1%) patients had stage 3B disease. All patients received four courses of chemotherapy during the neoadjuvant period. As the neoadjuvant chemotherapy regimen, 27 (47.4%) patients received cisplatin + paclitaxel, 11 (19.3%) patients received cisplatin + gemcitabine, 16 (28.1%) patients received cisplatin + docetaxel, and three (5.2%) patients received other regimens. Regarding radiological response rates, complete response was observed in three (5.3%) patients, partial response in 40 (70.2%) patients, and stable disease in five (8.8%) patients. After NAT, 15 (26.3%) patients were operated (pneumonectomy=26.7%, lobectomy=73.3%), 28 (49.1%) patients received chemoradiotherapy, and 14 (24.6%) patients were continued on palliative chemotherapy due to not being suitable for a definitive approach. Pathological complete response was observed in one (6.7%) of the 15 patients who were operated. The most common site of recurrence or metastasis after the post-NAT modality was the lung (59.1%), which was followed by the brain (22.7%) as the second most common site (Table 1).

**Table 1:** General characteristics of patients

	All patients, n=57(%)
Age (median, range) yr	58 (30-75)
Gender	
Male	52 (91.2)
Female	5 (8.8)
Smoking	
No	2 (3.5)
Yes	55 (96.5)
Pathological subtype	
Adenocarcinoma	19 (33.3)
Squamous cell carcinoma	38 (66.7)
Stage	
3A	41 (71.9)
3B	16 (28.1)
Neoadjuvant regimen	
Cisplatin + paclitaxel	27 (47.4)
Cisplatin + docetaxel	16 (28.1)
Cisplatin + gemcitabine	11 (19.3)
Others	3 (5.2)
Radiological response	
Complete response	3 (5.3)
Partial response	40 (70.2)
Stable disease	5 (8.8)
Progressive disease	9 (15.8)
Pathological response (n=15)	
Complete response	1 (6.7)
Partial response	14 (93.3)
Post-neoadjuvant treatment option	
Chemotherapy	14 (24.6)
Chemoradiotherapy	28 (49.1)
Surgery	15 (26.3)
Type of surgery (n=15)	
Lobectomy	11 (73.3)
Pneumonectomy	4 (26.7)
Area of recurrence or metastasis (n=44)	
Lung	26 (59.1)
Liver	1 (2.3)
Brain	10 (22.7)
Bone	7 (15.9)

When the distribution of the patients in the chemotherapy, chemoradiotherapy, and surgery arms was evaluated in terms of their general characteristics, histological subtypes, stages, received treatments, and neoadjuvant response rates; a history of smoking was more common in the surgery and chemoradiotherapy arms compared with the chemotherapy arm (p=0.04). The rate of patients that achieved a good response with NAT was higher in the surgery arm compared with the other two treatment arms (p=0.02). The three arms showed a similar distribution of patients with regard to the other characteristics (Table 2).

**Table 2:** Distribution of patients according to treatment modality after neoadjuvant therapy *One-way analysis of variance (ANOVA), ** Kruskal Wallis test

	All patients	Chemotherapy	Chemoradiotherapy	Surgery	P-value
Age (median, range) yr	58 (30-75)	59 (46-72)	59 (44-75)	57 (30-69)	0.39*
Gender					0.36**
Male	52 (91.2)	12 (85.7)	25 (89.3)	15 (100)	
Female	5 (8.8)	2 (14.3)	3 (10.7)	0 (0)	
Smoking					0.04**
No	2 (3.5)	2 (14.3)	0 (0)	0 (0)	
Yes	55 (96.5)	12 (85.7)	28 (100)	15 (100)	
Pathological subtype					0.65**
Adenocarcinoma	19 (33.3)	6 (42.9)	9 (32.1)	4 (26.7)	
Squamous cell carcinoma	38 (66.7)	8 (57.1)	19 (67.9)	11 (73.3)	
Stage					0.30**
3A	41 (71.9)	10 (71.4)	18 (64.3)	13 (86.7)	
3B	16 (28.1)	4 (28.6)	10 (35.7)	2 (13.3)	
Neoadjuvant regimen					0.45**
Cisplatin + paclitaxel	27 (47.4)	8 (57.2)	10 (35.7)	9 (60)	
Cisplatin + docetaxel	16 (28.1)	1 (7.1)	4 (14.3)	6 (40)	
Cisplatin + gemcitabine	11 (19.3)	4 (28.6)	12 (42.9)	0 (0)	
Others	3 (5.2)	1 (7.1)	2 (7.1)	0 (0)	
Radiological response					0.002**
Complete response	3 (5.3)	0 (0)	2 (7.1)	1 (6.7)	
Partial response	40 (70.2)	7 (50)	19 (67.9)	14 (93.3)	
Stable disease	5 (8.8)	1 (7.1)	4 (14.3)	0 (0)	
Progressive disease	9 (15.8)	6 (42.9)	3 (10.7)	0 (0)	

**Figure 1:**
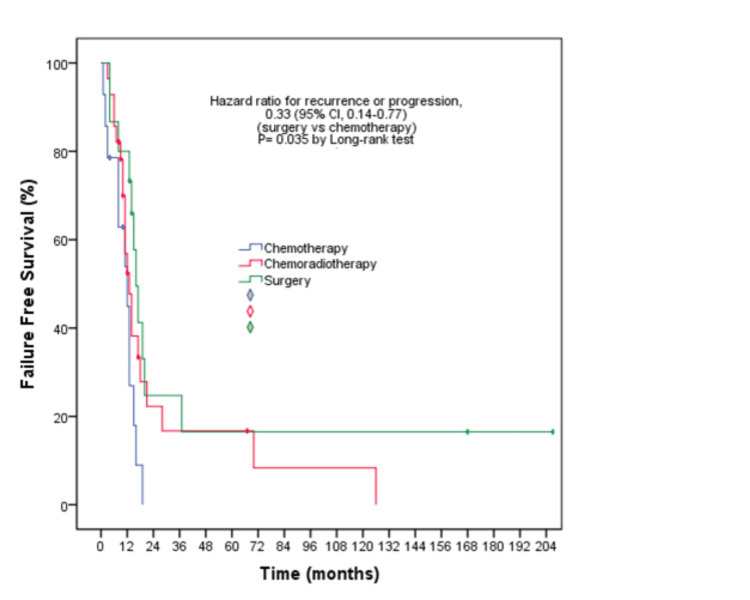
Failure-free survival outcomes according to post-neoadjuvant treatment modality

In the investigation of the factors influencing FFS in survival analyses, post-NAT modalities were found to show a statistically significant difference in the multivariate analysis. When the factors influencing OS were evaluated with univariate and multivariate analyses, the post-NAT treatment modalities were found to have a statistically significant effect on survival. FFS times were 12, 13, and 16 months in the chemotherapy, chemoradiotherapy, and surgery arms, respectively (log-rank p=0.035) (Figure 1).

**Figure 2:**
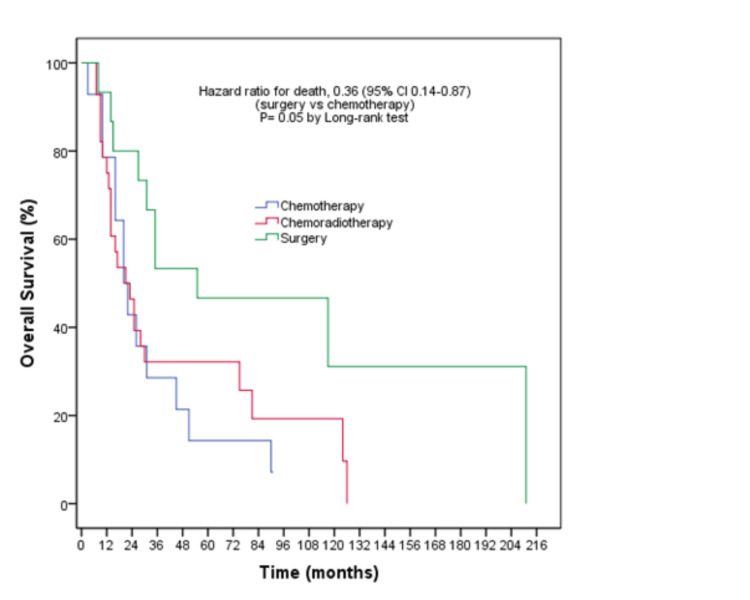
Overall survival outcomes according to post-neoadjuvant treatment modality

FFS was statistically longer in those who underwent surgery (Hazard ratio (HR): 0.33, 95% CI: 0.14-0.77, (p=0.01)). OS times were 20, 21, and 55 months in the chemotherapy, chemoradiotherapy, and surgery arms, respectively (log-rank p=0.05) (Figure 2).

OS was longer in the arm undergoing surgery compared to other arms (HR: 0.36, 95% CI: 0.14-0.87, (p=0.02)) (Table 3).

**Table 3:** Univariate and multivariate analysis results for failure-free survival and overall survival (*) Reference category, HR: hazard ratio, CI: confidence interval, SCC: squamous cell carcinoma

	Failure-Free Survival						Overall Survival					
	Univariate analysis			Multivariate analysis			Univariate analysis			Multivariate analysis		
Parameters	HR	95% CI	P	HR	95% CI	P	HR	95% CI	P	HR	95% CI	P
Age	1.01	0.97-1.04	0.65				1.01	0.98-1.05	0.36			
Gender (Male*/Female)	0.73	0.26-2.08	0.56				0.77	0.27-2.18	0.63			
Smoking (No*/yes)	0.42	0.10-1.78	0.24				0.84	0.20-3.53	0.82			
Pathological subtype (Adeno*/SCC)	0.95	0.51-1.78	0.88				0.80	0.43-1.47	0.47			
Stage (3A*/3B)	1.10	0.80-1.52	0.53				1.09	0.78-1.51	0.59			
Neoadjuvant regimen			0.35						0.20			
Cisplatin + paclitaxel*		reference						reference				
Cisplatin + gemcitabine	1.28	0.61-2.68	0.50				0.95	0.48-1.88	0.88			
Cisplatin + docetaxel	0.66	0.30-1.44	0.30				0.46	0.19-1.10	0.08			
Post-neoadjuvant treatment option			0.04			0.03			0.06			0.05
Chemotherapy*		reference			reference			reference			reference	
Chemoradiotherapy	0.52	0.25-1.10	0.08	0.51	0.24-1.08	0.07	0.83	0.41-1.65	0.59	0.82	0.40-1.68	0.6
Surgery	0.34	0.14-0.81	0.01	0.33	0.14-0.77	0.01	0.37	0.15-0.89	0.02	0.36	0.14-0.87	0.02

Five-year survival rates for the chemotherapy, chemoradiotherapy, and surgery arms were 14.3%, 21.4%, and 40%, respectively. Survival times and rates are presented in Table 4.

**Table 4:** Survival rates and survival times according to groups

	Chemotherapy	Chemoradiotherapy	Surgery
One-year survival rate (%)	78.6	78.6	93.3
Three-year survival rate (%)	28.6	32.1	53.3
Five-year survival rate (%)	14.3	21.4	40
Failure-free survival (median, months)	12	13	16
Overall survival (median, months)	20	21	55

## Discussion

Previous studies have shown that NAT is effective in downstaging and can offer a survival benefit in locally advanced NSCLC [12-14]. Although chemoradiotherapy offers higher effectiveness than chemotherapy during the neoadjuvant period, it is only used in limited situations due to significant toxicity [15]. It has been understood that surgery subsequent to NAT is more favorable than surgery alone in stage IIIA (N0-2) NSCLC [5,6]. Our study involved patients in the locally advanced stage (IIIA-IIIB) who were considered suitable for surgical resection after NAT and all patients had received chemotherapy during the neoadjuvant period. There were no patients with superior sulcus tumors or thoracic wall invasion that would require neoadjuvant chemotherapy + radiotherapy. The patients were re-evaluated for surgical suitability after four cycles of therapy. Patients suitable for surgery were operated. The form of treatment recommended for patients not suitable for surgery in locally advanced NSCLC is concurrent chemoradiotherapy [16]. Accordingly, in our study, patients not suitable for surgery after NAT were given concurrent chemoradiotherapy. For patients who were not suitable for definitive modalities or showed progression, the treatment proceeded with palliative chemotherapy.

In NSCLC, surgery is the ideal option for resectable patients. Meanwhile, in a locally advanced stage, surgery is the most effective option after NAT in eligible patients. Downstaging from N2 to N0 can be achieved in approximately 27% to 55% of the patients with NAT. Survival is poorer in those with persistent N2 disease [17,18]. It is important that complete resection is ensured in these patients. It has been reported that complete resection can be achieved in 65% of the patients with NAT and that the median survival time is 27 months in these patients compared with 12 months in those in whom resection is not achieved [3]. Another study determined the response rate as 54%, the OS as 24 months, and the five-year survival rate as 21% in stage-IIIA N2 patients after NAT [19]. In the Cancer and Leukemia Group B (CALGB) study, an OS of 20.9 months and a three-year survival rate of 46% were observed in IIIA N2 patients who underwent resection after NAT [12]. In our study, the rate of patients who underwent post-NAT surgery was 26.3%. OS was 55 months and the five-year survival rate was 40% in patients who underwent surgery. Meanwhile, the median OS times were 20 months and 21 months in the chemotherapy and chemoradiotherapy arms, respectively. There was a statistically significant advantage in OS in the surgical arm compared with the other arms (p=0.02). When evaluated with regard to FFS, the surgery, chemoradiotherapy, and chemotherapy arms were associated with FFS times of 16, 13, and 12 months, respectively. FFS was higher in the surgical arm than in the other arms (p=0.01). Our survival outcomes were better in comparison to the literature. In operated patients, pathological complete response was observed in eight (8.8%) of the 90 patients who underwent thoracotomy in the Spanish Lung Cancer Group study [17]. In our study, pathological complete response was obtained in 6.7% (3/15) of the patients who underwent surgery. Our pathological complete response outcomes were consistent with the literature.

The standard treatment approach for unresectable NSCLC is concurrent chemoradiotherapy. In the Radiation Therapy Oncology Group (RTOG) 8808 study, radiotherapy after induction chemotherapy was shown to be more advantageous than radiotherapy alone [20]. Meanwhile, in a phase-III Japanese study, concurrent chemoradiotherapy was found to offer better survival outcomes than sequential chemotherapy and radiotherapy (16.5 vs 13.3 months) [21]. On the other hand, the CALGB 9534 study investigated the effectiveness of concurrent chemoradiotherapy after two sessions of induction paclitaxel + carboplatin therapy and it was reported to be feasible in stage-III NSCLC. This study determined the response rate as 58%, the three-year FFS as 15%, the OS as 15.1 months, the one-year survival rate as 65%, and the three-year survival rate as 27%. In our study, patients who were not suitable for the surgical modality after NAT but were suitable for the definitive approach were given concurrent chemoradiotherapy. This population constituted 49.1% (n=28) of our patients. In this arm, the median FFS was 13 months, the median OS was 21 months, and the one- and three-year survival rates were 78.6% and 32.1%, respectively. Our outcomes were more favorable in comparison to the rates reported in the literature. However, patients whose treatment proceeded with palliative chemotherapy and patients who received concurrent chemoradiotherapy had comparable progression-free survival (12 months vs 13 months) (HR; 0.51, 95% CI; 0.24-1.08, p=0.07) and OS (20 months and 21 months) (HR; 0.80, 95% CI; 0.40-1.68, p=0.6) times.

As the NAT regimen, cisplatin + docetaxel is an effective treatment regimen [10]. In a study by Kocak M, et al., the use of cisplatin + docetaxel in induction therapy before chemoradiotherapy was reported to offer better survival outcomes than cisplatin + gemcitabine therapy in stage-III NSCLC [11]. In our study, three different neoadjuvant chemotherapy regimens (cisplatin + paclitaxel/docetaxel/gemcitabine) were used. In the evaluation of the groups with respect to survival, the three chemotherapy regimens were not significantly different in terms of FFS or OS.

In studies evaluating the other factors that influence survival, response to NAT and complete resection were determined to be significant prognostic factors for survival in patients operated after neoadjuvant chemotherapy [22]. A study conducted by Remark R, et al. suggested that radiological and pathological response status, surgical resection, and tumor subtype affected survival and that the immune pattern could be a factor in predicting survival [23]. Studies that are investigating factors such as smoking and the programmed death-ligand 1 (PD-L1) level in the use of chemotherapy immunotherapy during the neoadjuvant period in consideration of the subgroup analyses of the KEYNOTE - 189 study are currently underway [24,25]. In our study, age, gender, smoking, stage IIIA-B status, histological subtype, and response status were not observed to predict survival. However, we determined that survival was statistically higher in patients who underwent surgery after NAT (Table 3). Response status did not predict survival in our study, because a single post-NAT modality was selected in other studies. In our study, patient groups receiving chemotherapy or chemoradiotherapy were also included along with those that were suitable for surgery after NAT. The distribution of the patients revealed that the majority of patients who had a good response were found in the surgical arm. Thus, the heterogeneity in the patient distribution may have contributed to this situation.

Previous studies have reported the most common site of recurrence or metastasis after the definitive approach as the lung, followed by brain metastases as the second most common [3,22]. In our study, the evaluation of the most common sites of recurrence or metastasis after neoadjuvant or definitive treatment identified the lung as the site of recurrence of first metastasis in the majority of patients, with a rate of 59.1%. Brain metastases were the second most common with a rate of 22.7%.

The limitations of our study

It was a single-center study with a retrospective design, the number of patients was small, and the patient population and treatment groups were heterogeneous.

## Conclusions

In this study, we investigated the relationship between treatment modalities and survival in patients started on NAT in locally advanced (IIIA-B) NSCLC. We determined that the most important indicator of survival was the patients achieving an operable state and that the patients undergoing surgery had a significant advantage in OS and FFS when compared with patients receiving chemoradiotherapy or palliative chemotherapy. Additionally, we observed that patients receiving chemoradiotherapy and those receiving chemotherapy had comparable survival outcomes.
